# A WO_3_–CuCrO_2_ Tandem Photoelectrochemical Cell for Green Hydrogen Production under Simulated Sunlight

**DOI:** 10.3390/molecules29184462

**Published:** 2024-09-20

**Authors:** Ana K. Díaz-García, Roberto Gómez

**Affiliations:** 1 Institut Universitari d’Electroquímica i Departament de Química Física, Universitat d’Alacant, Apartat 99, E-03080 Alicante, Spain; koridiaz@uv.mx; 2 Facultad de Bioanálisis, Universidad Veracruzana, Xalapa C.P. 91010, Mexico

**Keywords:** photoelectrochemical tandem cell, water splitting, ternary oxides, tungsten oxide, acidic electrolytes, bias free

## Abstract

The development of photoelectrochemical tandem cells for water splitting with electrodes entirely based on metal oxides is hindered by the scarcity of stable p-type oxides and the poor stability of oxides in strongly alkaline and, particularly, strongly acidic electrolytes. As a novelty in the context of transition metal oxide photoelectrochemistry, a bias-free tandem cell driven by simulated sunlight and based on a CuCrO_2_ photocathode and a WO_3_ photoanode, both unprotected and free of co-catalysts, is demonstrated to split water while working with strongly acidic electrolytes. Importantly, the Faradaic efficiency for H_2_ evolution for the CuCrO_2_ electrode is found to be about 90%, among the highest for oxide photoelectrodes in the absence of co-catalysts. The tandem cell shows no apparent degradation in short-to-medium-term experiments. The prospects of using a practical cell based on this configuration are discussed, with an emphasis on the importance of modifying the materials for enhancing light absorption.

## 1. Introduction

Developing carbon-free energy technologies that are not only efficient but also in line with the conservation of the natural environment is one of the most pressing current challenges for humanity [1]. In this context, photoelectrochemical water splitting is a promising way of, not only harvesting, but also storing solar energy through the production of H_2_ from H_2_O [2,3,4].

The key components of a photoelectrochemical water splitting device are the photoactive materials [5,6,7,8]. Among them, typical photoelectrodes belong to several families of binary and ternary metal oxides. In fact, n-type oxides, such as Fe_2_O_3_ [9], TiO_2_ [10], WO_3_ [11] and BiVO_4_ [12], and p-type oxides, such as Cu_2_O [13], CuFeO_2_ [14], CaFe_2_O_4_ [15], CuFe_2_O_4_ [16], LaFeO_3_ [17] and CuCrO_2_ [18], as well as other ternary oxides [19,20], have been investigated for their use as photoanodes and photocathodes, respectively, in water splitting. In this context, the difficulty of developing photocathodes for the water reduction reaction based on nontoxic earth-abundant elements and possessing high stability should be stressed [2,19]. In any case, some works about photoelectrochemical water splitting using tandem cells under simulated sunlight have been published for a number of years. Specifically, tandem cells for water splitting have been investigated since 1977 [21]. However, even when rather high efficiencies were obtained, some of the materials employed were expensive and/or unstable, which made the studied systems unviable for real applications. In this sense, we believe that tandem photoelectrochemical cells based on earth-abundant metal oxides should be further developed since they may lead to, not only an affordable price, but also a stability in contact with aqueous solutions higher than that of electrodes based on chalcogenides, arsenides or nitrides.

A preliminary work on the use of both n-type and p-type hematite electrodes for zero-bias water photoelectrolysis was published as early as 2006 [22]. Interestingly, the electrolyte used was a dilute sulfuric acid solution, which led to short-term stability of the electrodes, although it was sufficient for carrying out some measurements. Ishihara et al. [23] demonstrated the unassisted operation of a tandem photoelectrochemical cell for water splitting based on metal oxides: TiO_2_ was employed as a photoanode and CaFe_2_O_4_ as a photocathode. However, the fact that Pt was used as a conducting substrate (too expensive for real applications) together with the employment of alkaline media (which may suffer from carbonation) should be taken into account. A few years later, Sivula et al. [24] demonstrated the unassisted operation of a tandem cell employing BiVO_4_ as a photoanode and Cu_2_O as a photocathode. However, the electrolyte was buffered to pH 6, which, according to Lewis et al. [25,26], is not desirable in terms of efficiency or safety. Moreover, some elements used in the structure of the photocathode (i.e., Au and RuO_x_) were expensive for a scalable process. Photocurrents corresponding to a solar-to-hydrogen (STH) conversion efficiency of ca. 0.5% were found to decay over a time of some minutes. Subsequent studies have expanded the number of electrode materials and strategies for interface engineering. It is worth noting that some of the proposed configurations are only partially based on metal oxides, as they also include either chalcogenides [27,28] or silicon [29] in their electrode composition.

In this work, we demonstrate the successful operation of a WO_3_–CuCrO_2_ tandem photoelectrochemical cell for water splitting under simulated sunlight without the application of external bias. Importantly, the tandem cell showed stability over 3 h without significant degradation, not only in neutral but also in acidic electrolytes, which is a remarkable achievement when compared with similar cell configurations. As far as we know, this is the first tandem cell based on unprotected metal oxides that works in acidic media in a stable way. Such conditions preclude the use of other photocathodes such as those based on CaFe_2_O_4_ or CuFeO_2_. In addition, the favorable combination of electrodes and electrolyte proposed in this work could also be used for other artificial photosynthesis processes (i.e., the reduction of CO_2_). Admittedly, the conversion efficiency is low, mainly due to both the lack of adequate solar light absorption and fast recombination. The strategies to be followed to overcome these limitations are also briefly discussed.

## 2. Results and Discussion

### 2.1. Morphological Characterization of Thin-Film Electrodes

**Figure 1:**
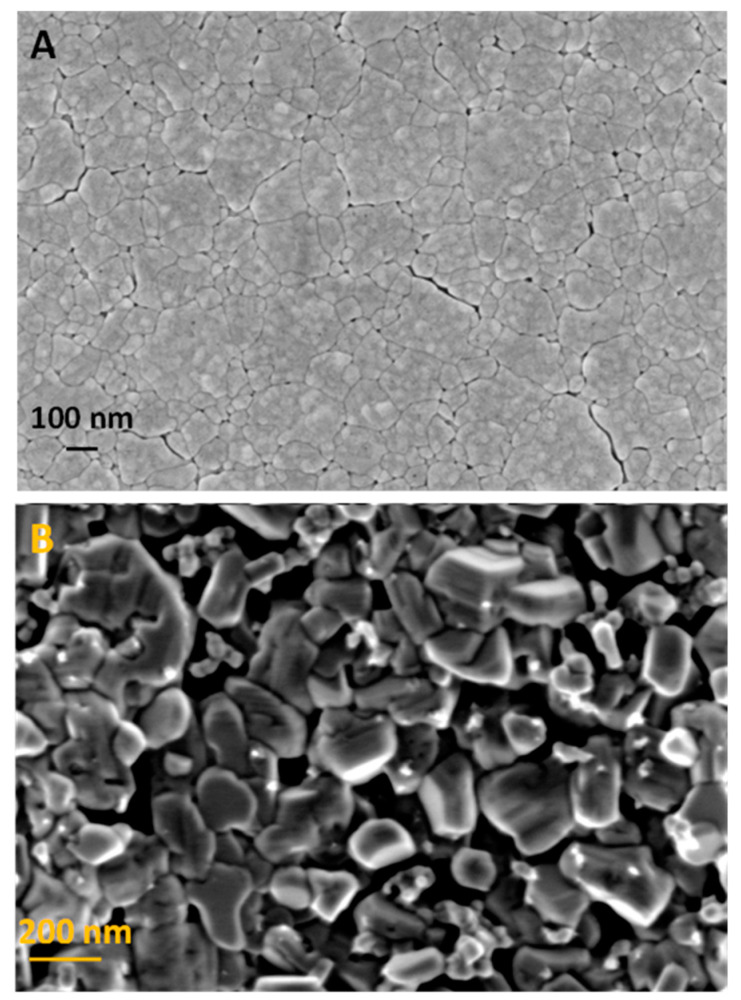
SEM images corresponding to (**A**) WO_3_ and (**B**) CuCrO_2_ thin-film electrodes.

The electrochemical and sol–gel synthetic methods employed for the preparation of the WO_3_ and CuCrO_2_ electrodes, respectively, yield rather compact thin films, as shown in Figure 1, particularly for the former. As observed, the grain size is significantly larger in the case of CuCrO_2_. This type of electrodes, as opposed to nanoporous ones, has the advantage that they can support a space charge layer, thus facilitating charge carrier separation [30].

It is worth noting that both electrode materials were thoroughly characterized in two previous papers from our laboratory devoted separately to WO_3_ [31] and CuCrO_2_ [18]. Spectroscopic and microscopic characterization (including TEM and AFM) of the WO_3_ electrodes revealed that the thin films were rather flat and consisted of monoclinic WO_3_ nanocrystals with a rather narrow size (diameter) distribution centered around 30 nm. Optical characterization revealed both transparency and a band gap of 2.65–2.7 eV. In the case of the CuCrO_2_ electrodes, XRD, microscopic and spectroscopic characterization showed that the thin films were a compact layer of rather large grains (size of around 150 nm) with a high degree of crystallinity corresponding to the pure delafossite phase. Importantly, optical measurements revealed high transparency and a band gap of 3.15 eV.

### 2.2. Photoelectrochemical Properties of WO_3_ Photoanodes and CuCrO_2_ Photocathodes

**Figure 2:**
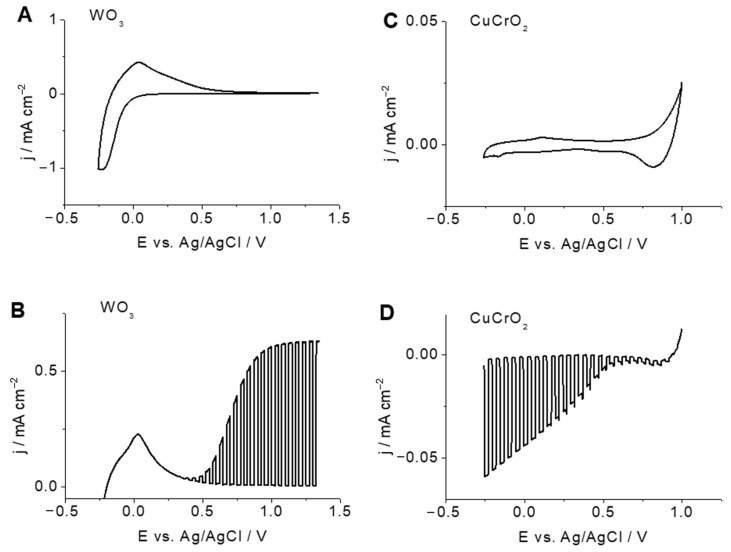
Cyclic voltammograms for (**A**) WO_3_ and (**C**) CuCrO_2_ electrodes in the dark in N_2_-purged 0.1 M HClO_4_. Scan rate 20 mV s^−1^. Linear scan voltammograms for (**B**) WO_3_ and (**D**) CuCrO_2_ electrodes in 0.1 M HClO_4_ purged with N_2_ under chopped simulated solar illumination (100 mW cm^−2^). Scan rate 5 mV s^−1^.

The photoelectrochemical behavior of the WO_3_ and CuCrO_2_ electrodes was first analyzed in a separate way in contact with perchloric acid solutions. The dark cyclic voltammogram (CV) for WO_3_ depicted in Figure 2A shows relatively large pseudo-capacitive currents in the low potential region (below 0.2 V), as expected for an n-type electrode material capable of adsorbing/intercalating protons. In addition, Figure 2B shows a corresponding linear scan voltammogram performed under chopped illumination. Anodic photocurrents start to appear close to the onset of the accumulation region. The voltammetric behavior, both in the dark and under illumination, shows that the material behaves as an n-type semiconductor. On the other hand, a CV for the CuCrO_2_ electrode is shown in Figure 2C, exhibiting small capacitive currents above 0.75 V_Ag/AgCl_, which correspond to an accumulation region. Figure 2D shows a linear scan voltammogram under chopped illumination for CuCrO_2_ electrodes, which is characterized by the existence of very small dark currents (in agreement with Figure 2C) together with significant cathodic photocurrents. The lack of significant dark currents is a first indication of the electrode stability in acidic electrolytes. The onset potential is located in this case at 0.90 V_Ag/AgCl_. Based on the CV results, CuCrO_2_ behaves as a p-type semiconductor. It should be noted that both photoelectrodes show stable responses, not only in close-to-neutral, but also in acidic electrolytes. The onset potential for the WO_3_ photoanode is located at 0.37 V (0.63 V vs. RHE), while significant photocurrents appear from 0.55 V (0.81 V vs. RHE) in the case of the CuCrO_2_ photocathode. Further details on the photoelectrochemical behavior of these electrodes can be found in our previous works [18,31].

**Figure 3:**
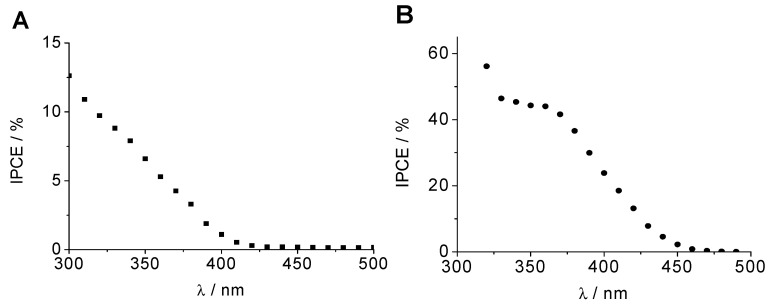
IPCE spectra for (**A**) CuCrO_2_ and (**B**) WO_3_ electrodes in 0.1 M HClO_4_ purged with N_2_ at 0.10 V_Ag/AgCl_ and 0.96 V_Ag/AgCl_, respectively.

IPCE spectra were also obtained at applied potentials of 0.10 and 0.96 V_Ag/AgCl_ for the CuCrO_2_ and WO_3_ electrodes, respectively, as shown in Figure 3. It should be mentioned that while the IPCE values are rather high for the WO_3_ photoanodes (almost 45% at 350 nm), they are relatively low for the CuCrO_2_ photocathodes (10% at 320 nm). This fact together with its wider band gap converts the CuCrO_2_ electrode into the limiting one in the tandem cell configuration.

In addition, a gas product analysis revealed that the Faradaic efficiency for hydrogen evolution was around 90% for the CuCrO_2_ photocathode, with an estimated relative error of 10% (Figure S1 in the SD shows the evolution of the electrode potential during photoelectrolysis). To our knowledge, this is the highest reported value for an oxide photocathode in the absence of co-catalysts or plasmonic effects at the surface of the photoelectrode [16,20,32].

**Figure 4:**
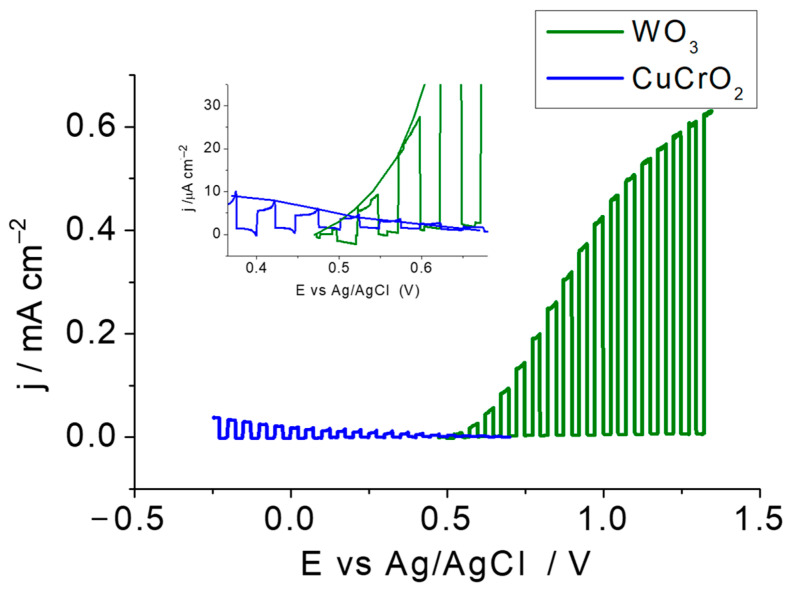
Linear scan voltammograms under chopped illumination for CuCrO_2_ (blue line) and WO_3_ (green line) electrodes in 0.1 M HClO_4_ purged with N_2_. Note that both cathodic and anodic currents are plotted as positive. The inset shows a detail of the region of curve crossing.

The expected tandem cell operating point at a short circuit was obtained as the crossing point of the absolute value of the photocurrent density vs. potential curves for both photoelectrodes, as shown in Figure 4, where the inset reveals that a very modest value of 5–10 μA cm^−2^ photocurrent can be expected from this tandem device without the application of bias and/or other optimization strategies.

The possibility of using these photoelectrodes in photoelectrochemical devices either as photoanodes or as photocathodes together with counter-electrodes working in the dark was first considered. In our measurements, Pt was used as a counter-electrode in both cases. In agreement with the results shown above, to obtain sizable photocurrents, a substantial bias (above 0.8 V) is needed in the case of WO_3_, while a smaller value (above 0.2 V) would be required for CuCrO_2_ (see SD, Figure S2). In any case, a bias-free device based on one photoelectrode is not possible.

### 2.3. Operation and Stability of the Tandem Cell

**Figure 5:**
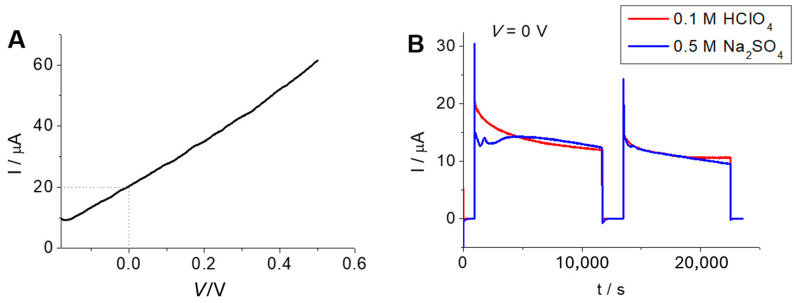
(**A**) Photocurrent vs. voltage curve (recorded at a scan rate of 2 mV s^−1^) for the tandem photoelectrochemical cell in 0.1 M HClO_4_ purged with N_2_ under illumination with a solar simulator (100 mW cm^−2^). (**B**) Comparative chronoamperometric curves for the tandem cell in either 0.1 M HClO_4_ or 0.5 M Na_2_SO_4_ (both purged with N_2_) under simulated solar illumination (100 mW cm^−2^) at zero bias. *V* = *E*_photoanode_ − *E*_photocathode_.

To check the functioning of the tandem photoelectrochemical system, a classical two-compartment H-type electrochemical cell equipped with fused silica windows was employed. Both electrodes were illuminated with the full output of a solar simulator at 1 sun (parallel illumination). The compartments were air-tight closed and purged with nitrogen prior to the measurements. They were separated by a Nafion membrane NM-117 (see SD, Figure S3). Figure 5A shows the photocurrent delivered by the device as a function of the applied bias (defined as the difference in potential between the photoanode and the photocathode) scanned at a rate of 2 mV s^−1^. As observed, the unbiased system delivers a photocurrent of 20 μA, which grows almost linearly with the applied bias. On the other hand, the stability of the cell was checked over a period of several hours, as shown in Figure 5B. After the decay of the photocurrent during the first hour, a remarkable stability was attained. In addition, with the aim of examining more thoroughly the efficiency and stability of the tandem photoelectrochemical cell, additional experiments were performed in close-to-neutral media. As observed, photocurrents and stability very similar to those observed in acidic media were found.

Despite the high value of Faradaic efficiency for hydrogen generation on the CuCrO_2_ electrode, photocurrents leading to only 0.006% solar-to-hydrogen (STH) conversion efficiency at a short circuit (zero bias) were determined [33]. Despite the very low STH conversion energy value, some key points should be highlighted. First, photoelectrochemical water splitting employing only oxide photoelectrodes is demonstrated for the first time in an acidic electrolyte. This overcomes the challenge of using either buffered or unbuffered near-neutral pH electrolytes [25,26], which could be unviable for real applications. It is worth noting that while using alkaline electrolytes may lead to carbonated media, working in acidic electrolytes avoids this disadvantage and, in addition, it minimizes ohmic drops in the electrolyte. Furthermore, the use of acidic electrolytes could be relevant in the potential application of the device for CO_2_ reduction without having to deal with the complexities derived from the formation of (bi)carbonate. Second, the photoelectrodes show remarkable short-to-medium-term stability (above 3 h), which is particularly relevant on account of the absence of any type of protective layer and the use of an acidic electrolyte. Third, even when no co-catalyst was used for the photocathode, a Faradaic efficiency for hydrogen evolution as high as 90% was determined, which is, as far as we know, the highest reported for a catalyst-free oxide photocathode. Fourth, both WO_3_ and CuCrO_2_ were synthesized by following a one-step wet procedure, without employing sophisticated physical methods, as for instance those needed in the case of Cu_2_O for increasing stability [34]. Finally, the electrodes were made of abundant elements, which adds potential applicability to the system. 

Admittedly, in comparison with other tandem photoelectrochemical cells of similar configuration [7,24,27], a very modest STH conversion efficiency (0.006%) was obtained. It should be noted, though, that neither the electrodes nor the device design was engineered to optimize the efficiency. Among the all-oxide photoelectrochemical tandem cells, as far as we know, this is the only one showing stable operation in an acidic electrolyte. In the case of alkaline electrolytes, a relatively stable behavior with an efficiency as high as 0.15% was reported for an iron oxide–copper bismuth oxide photoelectrochemical cell [35]. In contrast, STH efficiencies over 4% have been reported for close-to-neutral electrolytes by using sophisticated electrode architectures [36]. In terms of stability, the device presented here is in line with the state of the art, even when working with a highly acidic electrolyte.

**Figure 6:**
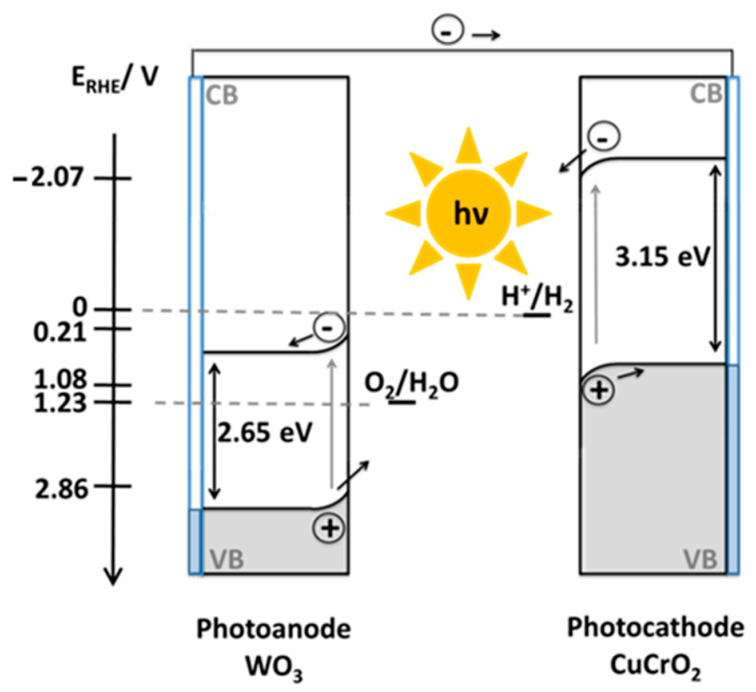
Sketch of the different potential levels relevant for the photoelectrodes and the electrolyte for the WO_3_/CuCrO_2_ photoelectrochemical tandem device.

There are several ways that one could pursue for increasing the device performance in addition to the application of an external bias. Some can be easily understood based on the sketch presented in Figure 6. Any surface modification of the WO_3_ electrode that could shift the flat band potential of WO_3_ toward less positive values would be beneficial. In clear connection with this, a TiO_2_ electrode would probably give comparable or even higher conversion efficiencies on account of its significantly lower flat band potential, even when only UV light would be useful. In addition, some strategies to tailor the interfaces of the CuCrO_2_ with passivating and/or extracting layers and with co-catalysts could be useful for increasing the relatively low IPCE values characterizing this material [14,37]. In any case, one of the main limitations in the STH conversion efficiency for the tandem device proposed here lies in the low solar light absorption capability of both electrode materials. Extensive doping could be one of the ways to address this problem [38,39,40,41,42].

## 3. Materials and Methods

### 3.1. Electrode Preparation

Sol–gel CuCrO_2_ photocathodes were prepared as previously described [18]. Cu(CH_3_COO)_2_∙H_2_O (purity 99.99%+, Sigma-Aldrich, Saint Louis, MO, USA) and Cr(NO_3_)_3_∙9H_2_O (purity 99%, Sigma-Aldrich, Saint Louis, MO, USA) were used as metal precursors. The precursor solution was spin-coated over FTO substrates (U-type, Asahi Glass Co., Japan) and the samples were annealed at 400 °C in air for 1 h. A total of four layers were deposited for synthesizing one electrode (i.e., the process was repeated 4 times to prepare one electrode). Finally, the samples were post-annealed at 650 °C in an N_2_ atmosphere. 

The electrodeposited WO_3_ photoanodes were prepared according to the procedure used by Luo and Hepel [43]. Briefly, tungsten powder (purity 99.99%, 12 μm, Sigma-Aldrich, Saint Louis, MO, USA) was dissolved in a concentrated hydrogen peroxide solution. Once the exothermic reaction was completed, ultrapure water and isopropanol (Merck p. a., Darmstadt, Germany) were added. This was the working solution used in a conventional three-electrode cell where the deposition was carried out. An Ag/AgCl/KCl(sat) electrode and a Pt wire were employed as a reference and as a counter-electrode, respectively, and a clean piece of FTO glass was used as a working electrode. A potential of −0.4 V vs. Ag/AgCl was applied to the FTO substrate for 30 min. Finally, the samples were post-annealed in air at 450 °C for 1 h.

### 3.2. Photoelectrochemical Measurements

Photoelectrochemical measurements were performed at room temperature using a cell based on a parallel illumination mode (a closed and sealed glass cell with two compartments separated by a Nafion^TM^ membrane (NM-117, DuPont, Wilmington, DE, USA) and a computer-controlled Autolab PGSTAT30 potentiostat (Metrohm Autolab B.V., Utrecht, The Netherlands). Depending on the experiment, an Ag/AgCl/KCl(sat) electrode and a Pt wire were employed as a reference and as a counter-electrode, respectively. Separate potentiostatic current–potential curves for both electrodes in this configuration were carried out in 0.1 M HClO_4_, which was prepared with ultrapure water and purged with N_2_ before the measurements. The illumination was carried out from the electrode substrate side by employing a solar simulator SUN 2000 (Abet Technologies, Milford, CN, USA). The light intensity was adjusted with a neutral density filter down to 100 mW cm^−2^ as measured with an optical power meter (Thorlabs model PM100D, Newton, NJ, USA). The illuminated area of each electrode was 2 cm^2^.

### 3.3. Tandem Cell Measurements

Currents through the cell were measured using two different working electrolytes: 0.5 M Na_2_SO_4_ and 0.1 M HClO_4_ under 1 sun illumination, as previously described. Perchloric acid was chosen as to avoid significant adsorption of the anion on the photoelectrodes. All the electrolytes were prepared with ultrapure water and purged with N_2_ before the experiments unless otherwise mentioned. The illuminated area of each electrode was 2 cm^2^.

### 3.4. Hydrogen Evolution Detection by Gas Chromatography

The analysis of hydrogen gas production was conducted using a Hewlett Packard 5890 gas chromatograph (Palo Alto, CA, USA) equipped with a thermal conductivity detector (TCD). The experiments were carried out in an airtight cell. The working electrode was a CuCrO_2_ photocathode (2 cm^2^ of illuminated area). In the counter-electrode compartment, a platinum sheet was placed, and in the reference compartment, an Ag/AgCl/KCl(sat) electrode was employed. The cell was filled with N_2_-purged 0.1 M HClO_4_, and prior to the experiments, its headspace was purged using a vacuum pump and filled with nitrogen gas several times. Samples of 300 μL were extracted from the headspace of the working electrode compartment.

## 4. Conclusions

In this work, we demonstrate that a WO_3_–CuCrO_2_ photoelectrochemical tandem cell is able to perform overall water splitting under simulated sunlight without the application of external bias. It is remarkable that the device works with strongly acidic electrolytes, which avoids complications linked to ohmic drops or electrolyte carbonation, showing remarkable short-to-medium-term stability (more than 3 h under operation without significant degradation). As far as we know, this is the first tandem cell with unprotected, co-catalyst-free metal oxide electrodes able to work in acidic media. In addition, it is worth noting that the Faradaic efficiency for hydrogen evolution is around 90% for the CuCrO_2_ photocathode without the use of co-catalysts, as far as we know the highest value reported to date for a co-catalyst-free oxide photocathode. Even though only a modest STH conversion efficiency was obtained for the tandem cell, some enhancement strategies apart from applying a bias could be pursued aiming to increase the solar light absorption, such as extensive doping of both electrode materials. In any case, this study illustrates the feasibility of overall water splitting working in acidic media with a tandem photoelectrochemical cell in which the electrodes are made of earth-abundant metal oxides by following simple and scalable procedures.

## Data Availability

The raw data supporting the conclusions of this article will be made available by the authors on request.

## Supplementary Materials

The following supporting information can be downloaded at https://www.mdpi.com/article/10.3390/molecules29184462/s1, Figure S1: Electrode potential vs. time curves during photoelectrolysis; Figure S2: Current density–voltage (j-V) curves; Figure S3: Photograph of the two-electrode PEC cell under operation.

## References

[1] Van de Krol R., Grätzel M. (2012). Photoelectrochemical Hydrogen Production.

[2] Prévot M.S., Sivula K. (2013). Photoelectrochemical tandem cells for solar water splitting. J. Phys. Chem. C.

[3] Walter M.G., Warren E.L., McKone J.R., Boettcher S.W., Mi Q., Santori E.A., Lewis N.S. (2010). Solar water splitting cells. Chem. Rev..

[4] Lewis N.S. (2016). Developing a scalable artificial photosynthesis technology through nanomaterials by design. Nat. Nanotechnol..

[5] Kang D., Kim T.W., Kubota S.R., Cardiel A.C., Cha H.G., Choi K.S. (2015). Electrochemical Synthesis of Photoelectrodes and Catalysts for Use in Solar Water Splitting. Chem. Rev..

[6] Harris-Lee T.R., Marken F., Bentley C.L., Zhang J., Johnson A.L. (2023). A chemist’s guide to photoelectrode development for water splitting—the importance of molecular precursor design. EES. Catal..

[7] Liu B., Wang S., Zhang G., Gong Z., Wu B., Wang T., Gong J. (2023). Tandem cells for unbiased photoelectrochemical water splitting. Chem. Soc. Rev..

[8] Moon C., Jung G., Min J., Shin B. (2024). Earth-Abundant Metal Oxides for Monolithic Tandem Photoelectrochemical Water Splitting Devices: Current Trends and Perspectives. ACS Mater. Lett..

[9] Lopes T., Andrade L., Le Formal F., Grätzel M., Sivula K., Mendes A. (2014). Hematite photoelectrodes for water splitting: Evaluation of the role of film thickness by impedance spectroscopy. Phys. Chem. Chem. Phys..

[10] Fujishima A., Honda K. (1972). Electrochemical photolysis of water at a semiconductor electrode. Nature.

[11] Chen X., Ye J., Ouyang S., Kako T., Li Z., Zou Z. (2011). Enhanced incident photon-to-electron conversion efficiency of tungsten trioxide photoanodes based on 3d-photonic crystal design. ACS Nano.

[12] Park Y., McDonald K.J., Choi K.-S. (2013). Progress in bismuth vanadate photoanodes for use in solar water oxidation. Chem. Soc. Rev..

[13] Luo J., Steier L., Son M.K., Schreier M., Mayer M.T., Grätzel M. (2016). Cu_2_O Nanowire Photocathodes for Efficient and Durable Solar Water Splitting. Nano Lett..

[14] Prévot M.S., Guijarro N., Sivula K. (2015). Enhancing the performance of a robust sol-gel-processed p-type delafossite CuFeO_2_ photocathode for solar water reduction. ChemSusChem.

[15] Díez-García M.I., Gómez R. (2016). Investigating Water Splitting with CaFe_2_O_4_ Photocathodes by Electrochemical Impedance Spectroscopy. ACS Appl. Mater. Interfaces.

[16] Díez-García M.I., Lana-Villarreal T., Gómez R. (2016). Study of Copper Ferrite as a Novel Photocathode for Water Reduction: Improving Its Photoactivity by Electrochemical Pretreatment. ChemSusChem.

[17] Díez-García M.I., Gómez R. (2017). Metal Doping to Enhance the Photoelectrochemical Behavior of LaFeO_3_ Photocathodes. ChemSusChem.

[18] Díaz-García A.K., Lana-Villarreal T., Gómez R. (2015). Sol-gel copper chromium delafossite thin films as stable oxide photocathodes for water splitting. J. Mater. Chem. A.

[19] Díez-García M.I., Gómez R. (2022). Progress in Ternary Metal Oxides as Photocathodes for Water Splitting Cells: Optimization Strategies. Solar RRL.

[20] Huang Q., Ye Z., Xiao X. (2015). Recent Progress in Photocathodes for Hydrogen Evolution. J. Mater. Chem. A.

[21] Ohashi K., McCann J., Bockris J.O. (1977). Stable photoelectrochemical cells for the splitting of water. Nature.

[22] Ingler W.B., Khan S.U.M. (2006). A Self-Driven p/n-Fe2O3 Tandem Photoelectrochemical Cell for Water Splitting. Electrochem. Solid State Lett..

[23] Ida S., Yamada K., Matsunaga T., Hagiwara H., Matsumoto Y., Ishihara T. (2010). Preparation of p-type CaFe_2_O_4_ photocathodes for producing hydrogen from water. J. Am. Chem. Soc..

[24] Bornoz P., Abdi F.F., Tilley S.D., Dam B., Van De Krol R., Grätzel M., Sivula K. (2014). A bismuth vanadate-cuprous oxide tandem cell for overall solar water splitting. J. Phys. Chem. C..

[25] Singh M.R., Papadantonakis K., Xiang C., Lewis N.S. (2015). An electrochemical engineering assessment of the operational conditions and constraints for solar-driven water-splitting systems at near-neutral pH. Energy Environ. Sci..

[26] Lichterman M.F., Sun K., Hu S., Zhou X., McDowell M.T., Shaner M.R., Richter M.H., Crumlin E.J., Carim A.I., Saadi F.H. (2016). Protection of inorganic semiconductors for sustained, efficient photoelectrochemical water oxidation. Catal. Today.

[27] Kim J.H., Kaneko H., Minegishi T., Kubota J., Domen K., Lee J.S. (2016). Overall photoelectrochemical water splitting using tandem cell under simulated sunlight. ChemSusChem.

[28] Bai Z., Zhang Y. (2017). A Cu_2_O/Cu_2_S-ZnO/CdS tandem photoelectrochemical cell for self-driven solar water splitting. J. Alloys Compd..

[29] Arunachalam M., Kanase R.S., Badiger J.G., Sayed S.A., Ahn K.-S., Ha J.-S., Ryu S.-W., Kang S.H. (2023). Durable bias-free solar Water-Splitting cell composed of n^+^ p-Si/Nb_2_O_5_/NiPt photocathode and W:BiVO_4_/NiCo(O-OH)_2_ photoanode. Chem. Eng. J..

[30] Osterloh F.F. (2013). Inorganic nanostructures for photoelectrochemical and photocatalytic water splitting. Chem. Soc. Rev..

[31] Monllor-Satoca D., Borja L., Rodes A., Gómez R., Salvador P. (2006). Photoelectrochemical Behavior of Nanostructured WO_3_ Thin-Film Electrodes: The Oxidation of Formic Acid. ChemPhysChem.

[32] Paracchino A., Brauer J.C., Moser J.E., Thimsen E., Graetzel M. (2012). Synthesis and characterization of high-photoactivity electrodeposited Cu_2_O solar absorber by photoelectrochemistry and ultrafast spectroscopy. J. Phys. Chem. C.

[33] Dotan H., Mathews N., Hisatomi T., Grätzel M., Rothschild A. (2014). On the solar to hydrogen conversion efficiency of photoelectrodes for water splitting. J. Phys. Chem. Lett..

[34] Paracchino A., Laporte V., Sivula K., Grätzel M., Thimsen E. (2011). Highly active oxide photocathode for photoelectrochemical water reduction. Nat. Mater..

[35] Du C., Yang J., Yang J., Zhao Y., Chen R., Shan B. (2018). An iron oxide -copper bismuth oxide photoelectrochemical cell for spontaneous water splitting. Int. J. Hydrogen Energy.

[36] Ye S., Shi W., Liu Y., Li D., Yin H., Chi H., Luo Y., Ta N., Fan F., Wang X. (2021). Unassisted Photoelectrochemical Cell with Multimediator Modulation for Solar Water Splitting Exceeding 4% Solar-to-Hydrogen Efficiency. J. Am. Chem. Soc..

[37] Prévot M.S., Li Y., Guijarro N., Sivula K. (2016). Improving charge collection with delafossite photocathodes: A host–guest CuAlO_2_/CuFeO_2_ approach. J. Mater. Chem. A.

[38] Lalanne M., Barnabé A., Mathieu F., Tailhades P. (2009). Synthesis and thermostructural studies of a CuFe_1−x_Cr_x_O_2_ delafossite solid solution with 0 ≤ x ≤ 1. Inorg. Chem..

[39] Taddee C., Kamwanna T., Amornkitbamrung V. (2016). Characterization of transparent superconductivity Fe-doped CuCrO_2_ delafossite oxide. Appl. Surf. Sci..

[40] Liew S.L., Subramanian G.S., Seng Chua C., Luo H.-K. (2016). Studies into the Yb-doping effects on photoelectrochemical properties of WO_3_ photocatalysts. RSC Adv..

[41] Zheng G., Wang J., Liu H., Murugadoss V., Zu G., Che H., Lai C., Li H., Ding T., Gao Q. (2019). Tungsten oxide nanostructures and nanocomposites for photoelectrochemical water splitting. Nanoscale.

[42] Costa M.B., de Araújo M.A., Tinoco M.V.D.L., Brito J.F.D., Mascaro L.H. (2022). Current trending and beyond for solar-driven water splitting reaction on WO_3_ photoanodes. J. Energy Chem..

[43] Luo J., Hepel M. (2001). Photoelectrochemical degradation of naphthol blue black diazo dye on WO_3_ film electrode. Electrochim. Acta.

