# Supplementary material for: A WO3–CuCrO2 Tandem Photoelectrochemical Cell for Green Hydrogen Production under Simulated Sunlight
Source: Molecules. 2024 Sep 20;29(18):4462. doi: 10.3390/molecules29184462 (PMC11434413; doi:10.3390/molecules29184462)
Supplement: Supplementary file 1 [file molecules-29-04462-s001.zip › molecules-3174615-supplementary.pdf]

Supplementary Material

A WO3 – CuCrO2 tandem photoelectrochemical cell for green hydrogen production under simulated sunlight

Ana Korina Díaz García and Roberto Gómez*

Departament de Química Física i Institut Universitari d'Electroquímica, Universitat d'Alacant. Apartat 99, 03080 Alicante, Spain

*Corresponding Author. Tel: +34 96 590 3748; email address: roberto.gomez@ua.es

1. Electrode potential vs. time curves during electrolysis.
2. Current density-voltage (j-V) curves.
3. Photograph of the two-electrode PEC cell under operation.

1. **Electrode potential vs. time curves during electrolysis**.

Chronopotentiometric measurements in Fig. S2 were performed during galvanostatic (photo)electrolysis show that the stability of the photocathode is not as good as that shown in the long-term chronoamperometric experiment. This could be linked to the fact that with an imposed current, the photocathode progressively shifts to potentials negative enough for damaging it.

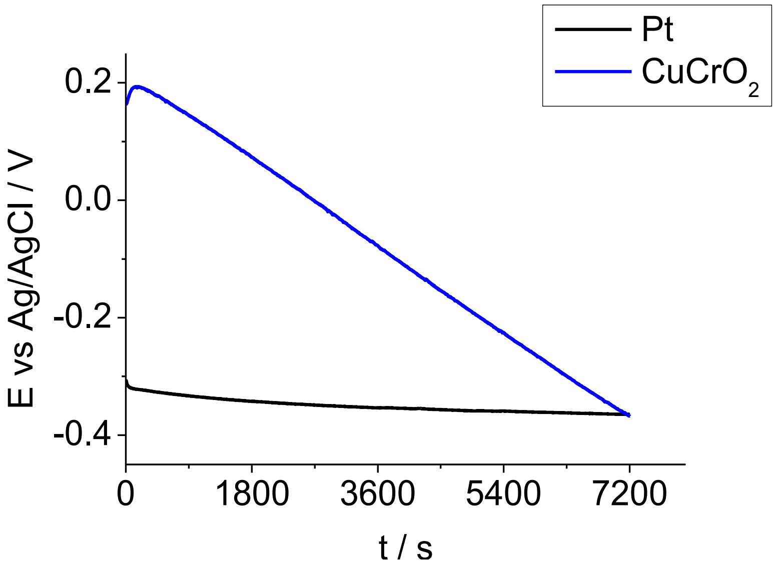

Figure S1. Electrode potential vs. time curves during (photo)electrolysis (applied current of 100 µA) for either an FTO/$CuCrO_2$ illuminated electrode (70 mW cm^{-2}) or a Pt sheet in the dark, both in N_2 purged 0.1 M $HClO_4$ electrolyte. The counter electrode was in both cases Pt foil.

2. Current density-voltage (j-V) curves.

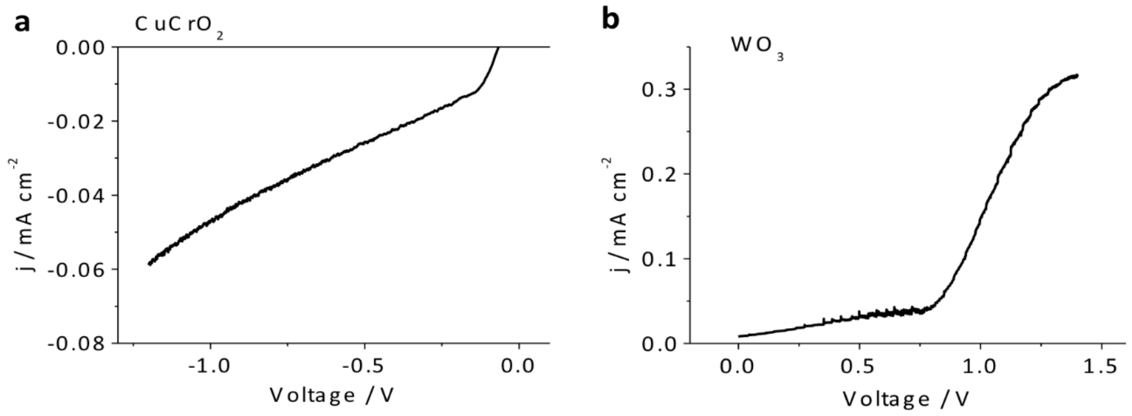

Figure S2. Current density-voltage curves for (a) a $CuCrO_2$ photocathode and (b) a WO_3 photoanode under simulated solar illumination (100 W cm^{-2}) in a two-compartment cell arrangement (working electrode: $CuCrO_2$ or WO_3, and counter electrode: Pt sheet). Scan rate 5 mV s^{-1}.

3. **Photograph of the two-electrode PEC cell under operation**.

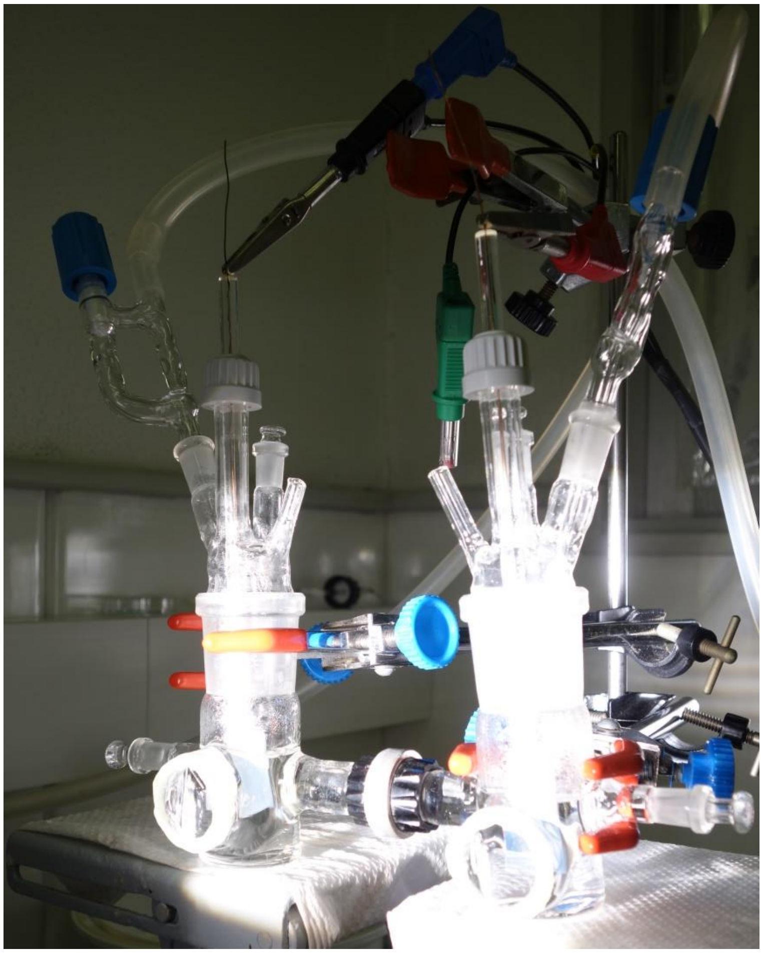

Figure S3. Picture of the two-electrode photoelectrochemical cell under operation.
